# Influence of college students’ professional identity on learning burnout: the mediating role of emptiness and the moderating role of career calling

**DOI:** 10.3389/fpsyg.2025.1737379

**Published:** 2026-01-08

**Authors:** Jianjun Ni, Weihang Liu, Qingting Zhou, Lian He, Yun Yang

**Affiliations:** 1 Department of Business, Southwest Jiaotong University Hope College, Chengdu, China; 2 School of Business Administration, Southwestern University of Finance and Economics, Chengdu, China; 3 Research Laboratory of Plastic and Burns Surgery, West China Hospital, Sichuan University, Chengdu, China

**Keywords:** career calling, college students, emptiness, learning burnout, professional identity

## Abstract

**Introduction:**

Existing research suggests that professional identity plays a critical role in shaping students’ learning attitudes and academic behaviors; however, the underlying mechanisms remain insufficiently understood. This study aims to examine the impact of professional identity on learning burnout among Chinese university students and to investigate the mediating role of a sense of emptiness and the moderating role of career calling.

**Methods:**

In this study, undergraduate students from three universities in Western China were recruited as research participants, and data were collected through validated standardized questionnaires. Correlation analysis, mediation testing, and moderation analysis were conducted to examine the relationships among the key variables.

**Results:**

The results revealed that (1) professional identity significantly negatively predicts learning burnout and that (2) emptiness partially mediates the relationship between professional identity and learning burnout, indicating that professional identity can reduce learning burnout by lowering the sense of emptiness. (3) Career calling had a significant positive moderating effect on the relationship between emptiness and learning burnout, suggesting that when students’ career calling is high, the positive impact of emptiness on learning burnout becomes more pronounced.

**Discussion:**

The findings further corroborated the negative association between professional identity and learning burnout. Moreover, the results revealed that emptiness served as a mediator linking professional identity to learning burnout, whereas career calling positively moderated the effect of emptiness on students’ learning burnout. These findings highlight the importance of strengthening students’ professional identity and career calling, as well as mitigating feelings of emptiness, in efforts to reduce learning burnout among university students.

## 1 Introduction

In the context of increasingly fierce social competition and increasing educational pressure, learning burnout has become prevalent among college students, with significant negative effects on academic performance and psychological well-being (Liu et al., 2023; Barbayannis et al., 2022). Learning burnout is a psychological syndrome closely related to chronic academic stress, typically manifested as emotional exhaustion, reduced interest in and enthusiasm for academic activities, increased feelings of alienation from peers and the learning environment, and a sense of helplessness and negativity resulting from a lack of achievement (Meier and Schmeck, 1985).

Previous research has shown that learning burnout not only directly impedes students’ academic development but also poses potential threats to their future career planning and professional growth (Mao and Liao, 2025). At an academic level, learning burnout leads to decreased learning motivation, poorer academic performance, and less efficient utilization of educational resources (Barbayannis et al., 2022). From a career development perspective, students who experience chronic burnout demonstrate lower levels of professional commitment and career identity, which may undermine career stability and adaptability (Pham Thi and Duong, 2024). More severely, learning burnout may reduce individuals’ psychological resilience and career adaptability, thereby increasing the risk of maladaptive behaviors such as internet addiction and social avoidance, thereby jeopardizing students’ mental health, learning quality, and long-term development (Wang et al., 2025; Oliveira and Marques, 2024; Chong et al., 2025).

Therefore, a systematic exploration of the influencing factors and underlying mechanisms of learning burnout among college students holds significant theoretical and practical value for fostering their psychological well-being and sustainable academic development. Numerous studies have indicated that among the various factors influencing learning burnout, psychological traits and cognitive variables play particularly crucial roles, with professional identity being among the core psychological resources (Chong et al., 2025). When college students possess a strong sense of professional identity, they tend to have clearer learning goals, a stronger sense of professional value, and higher levels of engagement and self-efficacy, all of which help to effectively mitigate learning burnout (Wang and Jiang, 2025; Monti et al., 2025). In contrast, students with a low level of professional identity are more prone to experience academic alienation and psychological emptiness (Qiu et al., 2023).

Although prior research generally indicates that professional identity can significantly reduce learning burnout among university students, the literature still has notable limitations in terms of elucidating its underlying mechanisms. Insufficient attention has been given to deeper affective factors—such as the loss of meaning and emotional depletion experienced by students in the learning process (Mannerström et al., 2024)—leaving the value-based pathways through which professional identity influences learning burnout insufficiently explored. Furthermore, although existing studies have acknowledged the positive role of career calling in variables such as learning engagement and burnout (Lian et al., 2021; Zhao et al., 2022), the discussion remains largely at the level of main effects. Limited research has investigated whether career calling, as a stable psychological resource, can function as a moderator within the “professional identity–sense of emptiness–learning burnout” mechanism.

Drawing on self-determination theory, this study incorporates emptiness as a mediating variable and career calling as a moderating variable to construct a mechanism model explaining how professional identity influences learning burnout among university students. Specifically, the mediating role of emptiness in the relationship between professional identity and learning burnout and the moderating effect of career calling on the “professional identity → emptiness → learning burnout” pathway are examined. By doing so, both the underlying mechanisms and boundary conditions through which professional identity shapes students’ learning burnout are identified in this study.

This research contributes to the literature by addressing the underexplored issue of meaning deprivation, thereby offering a more fine-grained understanding of the psychological processes linking professional identity to learning burnout. Validated scales will be used to construct the survey instrument, with current university students serving as participants. The data are analyzed using SPSS and the PROCESS macro to empirically test the proposed model. The findings are expected to enhance our understanding of the factors and mechanisms influencing learning burnout and to provide practical implications for promoting student development in higher education.

## 2 Theoretical framework and research hypotheses

### 2.1 Relationship between professional identity and learning burnout

Professional identity refers to a psychological process in which individuals, drawing upon their understanding of their academic discipline, develop emotional attachment and value resonance toward it, which is reflected behaviorally through active engagement and continuous learning (Qin, 2009). As a core component of self-identity, professional identity plays a critical role in shaping students’ attitudes and behaviors toward learning (Reid et al., 2008). Existing research indicates that students with a stronger level of professional identity tend to demonstrate more positive learning attitudes, stronger self-directed learning abilities, and greater engagement in academic activities (Liao et al., 2024). Such adaptive learning states stem from students’ deep understanding of the meaning, value, and future prospects associated with their major, which in turn enhances their learning motivation and overall learning experience (Martela et al., 2021).

Self-determination theory provides a foundational lens for understanding how professional identity shapes students’ learning experiences (Ryan and Deci, 2000). The theory posits that students’ learning motivation depends on the extent to which three basic psychological needs—autonomy, competence, and relatedness—are fulfilled (Ryan and Deci, 2000). When students develop a strong sense of identification with their major, they are more likely to perceive learning as a self-endorsed choice (autonomy), believe that they are capable of mastering academic tasks (competence), and feel connected to their professional community (relatedness). The satisfaction of these needs fosters stable intrinsic motivation, promotes sustained engagement, and reduces emotional exhaustion driven by external pressures. Conversely, when students lack professional identity, they are more prone to experiencing amotivation, diminished meaning, and other negative psychological states, thereby increasing their risk of learning burnout.

Previous research has demonstrated that a high level of professional identity can significantly influence students’ cognitive patterns, emotional experiences, and learning behaviors (Chen et al., 2025). When college students have a strong sense of professional identity, they are inclined to evaluate their abilities more positively, enhance their self-efficacy and learning motivation, and thereby buffer the adverse effects of academic pressure (Zou et al., 2024). Empirical evidence further indicates that students with stronger professional identity demonstrate greater academic engagement, better learning efficacy, more positive emotional experiences, and significantly lower levels of learning burnout (Chen et al., 2025; Zou et al., 2024).

These findings highlight the pivotal role played by professional identity in alleviating learning burnout by reinforcing learning motivation and facilitating positive emotional experiences.

*Hypothesis H1*: Professional identity has a significant negative effect on college students’ learning burnout.

### 2.2 Mediating role of emptiness

Emptiness is a negative psychological experience stemming from a deficit in personal meaning and value; it manifests as inner hollowness, aimlessness, and emotional indifference and is often accompanied by maladaptive behaviors and psychological distress (Liang, 2013). As an internalized form of psychopathology, emptiness has been shown to impair individuals’ appetite and attentional quality and to diminish their interest in previously preferred activities (Castro et al., 2018). It may also be accompanied by self-directed harmful behaviors and, in more severe cases, can lead to social dysfunction, depression, and other critical mental health problems (Miller et al., 2018; Castro et al., 2018; Harford et al., 2019). Among university students, emptiness is a persistent negative emotional experience that can trigger various adverse emotions and maladaptive behaviors, thereby undermining their adjustment to campus life (Tarrant et al., 2006). Conversely, difficulties in academic and social adjustment may further induce feelings of helplessness, alienation, and diminished self-worth, reinforcing the experience of emptiness (Liang, 2013). Previous studies have primarily regarded emptiness as a concomitant symptom of emotional problems such as depression and anxiety (Hang et al., 2025), with limited exploration of its role in academic psychological processes.

From an existentialist perspective, ambiguous life goals and a lack of personal meaning constitute the core psychological foundations of emptiness (Çevik et al., 2020; Cho and Jang, 2021). When students’ academic majors align well with their personal interests and expectations, a strong professional identity motivates them to actively explore disciplinary knowledge and engage in practical activities, allowing them to experience learning as meaningful and fulfilling (Messerer et al., 2024; Lu et al., 2025). Conversely, students with low professional identity often lack goal orientation and emotional connection, leading to confusion and helplessness in the learning process, which perpetuates persistent feelings of emptiness (Wu et al., 2024; Zou et al., 2024).

Furthermore, self-determination theory posits that human behavior is driven by intrinsic motivation, extrinsic motivation, and amotivation (Ryan and Deci, 2000). When individuals act on intrinsic motivation—behaviors aligned with their own interests and values—they experience greater satisfaction and autonomy, which in turn reduces depressive states such as emptiness (Liang, 2013). Within this theoretical framework, professional identity is conceptualized as an important form of intrinsic motivation in university students’ learning and development (Tarrant et al., 2006). A higher level of professional identity reflects students’ positive emotional acceptance, value endorsement, and goal alignment with their chosen major, making them more likely to perceive learning as an activity coherent with the self. Such identification not only sustains long-term learning interest but also enhances students’ sense of autonomy and competence—that is, the belief that they can effectively master relevant knowledge and skills. When these basic psychological needs are fulfilled, students experience strengthened intrinsic motivation, thereby forming a positive cycle of learning engagement and emotional well-being. Consequently, higher professional identity promotes greater learning interest, autonomy, and competence, which helps reduce the emergence of emptiness and related negative emotions.

*Hypothesis H2a*: Professional identity has a significant negative effect on emptiness.

Moreover, chronic emptiness can deplete individuals’ psychological resources and self-regulatory capacity. This often leads to compensatory behaviors, including internet addiction and excessive smartphone use, which serve as maladaptive strategies for regaining emotional balance and ultimately exacerbate learning burnout (Frankl, 1984; Gu et al., 2023; Yang et al., 2024; Ye et al., 2025). Research on adolescent internet addiction has shown a positive correlation between the level of internet addiction and learning burnout (Wang et al., 2007). Studies examining the relationship between university students’ sense of emptiness and smartphone dependence have similarly reported a positive association (Gao et al., 2020). As a maladaptive compensatory behavior, excessive smartphone use can exacerbate negative emotions, reduce academic self-efficacy, and deplete cognitive resources when students encounter learning difficulties, thereby increasing the likelihood of learning burnout (Wang et al., 2025). These findings suggest that maladaptive compensatory behaviors arising from emptiness are directly or indirectly linked to learning burnout. Taken together, students with low professional identity are less emotionally engaged with their major and more likely to experience confusion and a lack of meaning in learning, which can lead to emptiness, and the maladaptive behaviors associated with emptiness may ultimately trigger learning burnout. On the basis of this rationale, the following hypotheses are proposed:

*Hypothesis H2b*: Emptiness has a significant positive effect on learning burnout.

*Hypothesis H2c*: Emptiness has a significant mediating effect on the relationship between professional identity and learning burnout.

### 2.3 Moderating role of career calling

Career calling refers to an individual’s sense of enthusiasm, identification, and responsibility toward the meaning and value of his or her future profession. It represents a stable and intrinsically motivated orientation toward one’s career (Duffy and Dik, 2013). A defining characteristic of individuals with a strong sense of calling is their action orientation; they are motivated to actively engage in activities that align with their calling (Elangovan et al., 2010). When individuals hold a calling toward a particular profession, they tend to highly value and perceive significance in that career, which drives them to devote substantial effort to related professional activities (Dobrow and Tosti-Kharas, 2011; Hirschi, 2012; Serow, 1994). The university period represents a critical stage of career preparation, and students with a strong sense of career calling are likely to actively participate in professional development activities aimed at acquiring the skills necessary to fulfill their calling. Furthermore, research among university students has indicated that those with strong career calling often demonstrate greater purpose, stronger career planning behaviors, and heightened recognition of the relevance between current academic pursuits and future professional goals (Duffy and Dik, 2013).

Grounded in self-determination theory (Ryan and Deci, 2000), career calling can be conceptualized as a highly self-determined form of motivation that fulfills individuals’ basic psychological needs for autonomy, competence, and relatedness, thereby fostering greater learning engagement and psychological resilience (Allan et al., 2016; Wen et al., 2023). Empirical studies indicate that career calling can buffer against the sense of meaninglessness and emptiness that may arise from low levels of professional identity (Lu et al., 2025) and plays a positive moderating role in the learning process. For instance, among preservice teachers, higher levels of career calling have been shown to significantly predict learning motivation and the use of cognitive strategies, thereby effectively reducing the risk of learning burnout (Shang et al., 2022). Moreover, students with a strong sense of calling tend to actively engage in learning activities that align with their abilities and interests, which helps mitigate the academic exhaustion associated with feelings of emptiness and meaninglessness (Wells et al., 2023).

Therefore, career calling serves as a protective psychological resource that helps alleviate the negative impact of emptiness on learning burnout.

*Hypothesis H3*: Career calling acts as a negative moderator between emptiness and learning burnout. The strength of the positive relationship between these two variables decreases when students possess a stronger sense of career calling.

**Figure 1:**
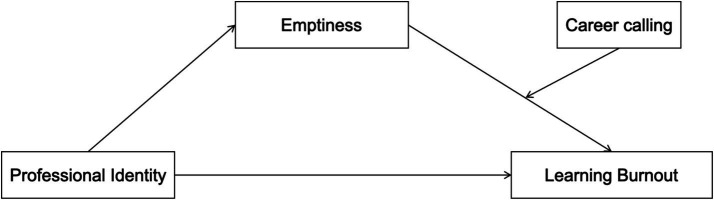
Theoretical model.

In summary, the findings of this study suggest an integrated model in which professional identity is the independent variable, learning burnout is the dependent variable, emptiness serves as the mediating variable, and career calling functions as the moderating variable. This framework aims to explore the underlying psychological mechanisms and boundary conditions of learning burnout among university students (see Figure 1).

## 3 Research participants and methods

### 3.1 Research participants

In this study, undergraduate students from three universities located in western China were selected as the research subjects. Data were collected using the online survey platform Questionnaire Star^1^ from May 19 to June 13, 2025. A random sampling method was adopted. The questionnaires were distributed through a combination of offline and online channels: offline administration involved inviting students to complete the survey between classes, while online distribution utilized teacher and peer networks to disseminate the electronic survey link via QQ, WeChat, and email to currently enrolled undergraduates. A total of 663 questionnaires were collected. After those with missing responses, identical or patterned answers, careless or perfunctory completion, and unusually short response times were excluded, 513 valid questionnaires were retained, yielding an effective response rate of 77.38%.

The demographic characteristics of the sample are presented in Table 1.

**Table 1:** Demographic characteristics of the sample (*N* = 513).

Variable	Category	Frequency (n)	Percentage (%)
Gender	Male	157	30.6
	Female	356	69.4
Grade	Freshman	181	35.3
	Sophomore	157	30.6
	Junior	157	30.6
	Senior	18	3.5
Place of origin	Urban area	179	34.9
	County-level city	96	18.7
	Township	75	14.6
	Rural area	163	31.8
Major category	Humanities and social sciences	216	42.1
	Science and engineering	273	53.2
	Arts and physical education	24	4.7

In this study, the minimum required sample size was estimated using two approaches. First, Fritz and MacKinnon’s (2007) Monte Carlo simulation indicated that testing a mediation effect with a medium effect size requires 200–400 participants to achieve 80–90% statistical power. Second, Kline (2015) recommended that regression-based and mediation models should include at least 200–300 participants. Considering potential sample loss due to incomplete or invalid questionnaires, the target sample size for this study was set at 500 participants.

The inclusion criteria for this study were as follows: (1) being a currently enrolled undergraduate student; (2) having the ability to independently comprehend and complete the questionnaire; and (3) providing informed consent voluntarily. The exclusion criteria were (1) unwillingness or inability to complete the questionnaire; (2) responses exhibiting obvious patterns or uniformity; and (3) questionnaires with missing data.

### 3.2 Measurement of variables

The primary variables measured in this study included professional identity, career calling, college students’ sense of meaninglessness, and learning burnout. All the scales employed in this study were developed by Chinese scholars within the Chinese linguistic and cultural context. Their item descriptions are consistent with Chinese students’ language habits and comprehension levels, thereby enabling more accurate assessment of the target population. These measurement scales have demonstrated satisfactory reliability and validity across previous empirical studies. Each scale employed a five-point Likert scoring system, with scores indicating the extent to which respondents agreed that the statement applied to them. A score of 1 represents “strongly disagree,” whereas a score of 5 represents “strongly agree.”

The Professional Identity Scale adopted Qin (2009) research questionnaire, comprising 23 items across four dimensions: cognitive, emotional, behavioral, and appropriateness. A 5-point rating scale was used, with higher scores indicating stronger professional identity. Example items include statements such as “I understand the competence requirements of my major” and “I am aware of the employment prospects associated with my major.” In the present study, the scale demonstrated excellent internal consistency, with a Cronbach’s *α* coefficient of 0.951, indicating highly reliable measurement performance.

Learning burnout was assessed using the College Student Learning Burnout Scale developed by Lian et al. (2005), which consists of 20 items categorized into three dimensions: dejection, misconduct, and reduced sense of achievement. Higher scores indicate higher levels of learning burnout. Example items include statements such as “I have my own learning strategies and plans, and I am able to carry them out” and “I feel that what I am learning is useless.” In the present study, the internal consistency of the scale was good, with a Cronbach’s *α* coefficient of 0.858, indicating satisfactory reliability.

Career calling was measured using the Career Calling Scale developed by Zhang et al. (2014), which includes 11 items and four dimensions: altruistic contribution, guidance, meaning, and value. Higher scores represent a stronger sense of career calling. Example items include statements such as “I want my future work to contribute to society” and “I aspire to engage in a profession that benefits others.” In the present study, the scale demonstrated good internal consistency, with a Cronbach’s α coefficient of 0.871, indicating satisfactory reliability.

Sense of emptiness was assessed using the College Students’ Emptiness Scale developed by Liang (2013). The scale contains 11 items distributed across four dimensions: sense of value, emotional experience, volitional freedom, and negative behavior. Higher scores reflect a stronger sense of emptiness. Example items include statements such as “I am unable to feel a sense of personal worth” and “Even when I have free time, I have no desire to study.” In the present study, the internal consistency of the scale was excellent, with a Cronbach’s α coefficient of 0.946, indicating high reliability.

### 3.3 Data analysis

All the statistical analyses were performed using SPSS version 31.0. Descriptive statistics were calculated using the frequency (n) and percentage (%) used for categorical variables and the mean ± standard deviation (M ± SD) used for continuous variables.

Hayes’ PROCESS macro (Version 3.5) was utilized to test the hypothesized mediation and moderation effects. Specifically, a mediation analysis was conducted to examine the indirect effects of professional identity on learning burnout via a sense of emptiness, and a moderation analysis was performed to examine the buffering effect of career calling.

## 4 Data analysis and hypothesis testing

### 4.1 Test for common method bias

To assess the potential common method bias in the data, Harman’s single-factor test was conducted on all measurement items using unrotated principal component analysis. The results indicated that 12 factors had eigenvalues greater than 1, and the first factor accounted for 32.09% of the total variance, which was below the critical threshold of 40%. Therefore, the results suggest that no significant common method bias was present in the data (Hair et al., 1998).

### 4.2 Descriptive statistics and correlation analysis

As shown in Table 2, the relationships among professional identity, career calling, a sense of emptiness, and learning burnout among college students were as follows: Professional identity was significantly negatively correlated with a sense of emptiness (*r* = −0.609, *p* < 0.01). Professional identity was significantly positively correlated with career calling (*r* = 0.632, *p* < 0.01). Professional identity was significantly negatively correlated with learning burnout (*r* = −0.673, *p* < 0.01). Sense of emptiness was significantly negatively correlated with career calling (*r* = −0.417, *p* < 0.01). Sense of emptiness was significantly positively correlated with learning burnout (*r* = 0.745, *p* < 0.01). Career calling was significantly negatively correlated with learning burnout (*r* = −0.461, *p* < 0.01).

**Table 2:** Descriptive statistics and correlation matrix of key variables (*N* = 513).

Variable	*M*	SD	1	2	3	4
1. Professional identity	3.60	0.65	—			
2. Sense of emptiness	2.57	0.74	−0.609**	—		
3. Career calling	3.58	0.61	0.632**	−0.417**	—	
4. Learning burnout	2.82	0.55	−0.673**	0.745**	−0.461**	—

M, mean; SD, standard deviation. *p < 0.05; **p < 0.01.

These findings preliminarily indicate that professional identity and career calling may play protective roles in mitigating learning burnout, whereas a stronger sense of emptiness is associated with higher levels of burnout.

### 4.3 Test of the mediating effect of sense of emptiness

After controlling for gender, grade level, major, and place of origin, professional identity was used as the independent variable, learning burnout was used as the dependent variable, sense of emptiness was used as the mediating variable, and career calling was used as the moderating variable. The mediating effect was tested using Model 4 of the PROCESS macro for SPSS (Hayes, 2013). Following Hayes’s bootstrap approach, bias-corrected 95% confidence intervals were calculated to evaluate the significance of the indirect effect. All the continuous variables were mean centered prior to the analysis.

**Figure 2:**
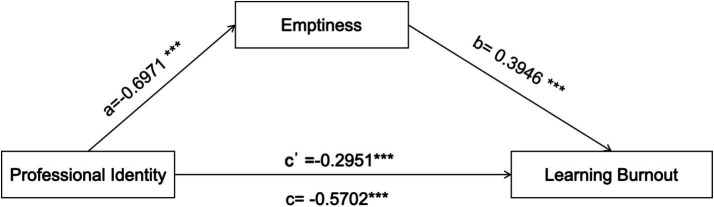
Path diagram illustrating the mediating role of sense of emptiness in the relationship between professional identity and learning burnout. ***Indicates the statistical significance level, *p* < 0.001.

Following the procedure proposed by Wen and Ye (2014), the mediating role of sense of emptiness between professional identity and learning burnout was further examined (see Figure 2). The results (see Table 3) indicated that sense of emptiness exerted a significant partial mediating effect on the relationship between professional identity and learning burnout.

**Table 3:** Decomposition of the effects of professional identity, a sense of emptiness, and learning burnout.

Effect type	Effect value	SE	LLCI	ULCI	Proportion
Total effect	−0.5702	0.0277	−0.6247	−0.5157	—
Direct effect	−0.2951	0.0287	−0.3515	−0.2387	51.75%
Indirect effect	−0.2751	0.0243	−0.3225	−0.2279	48.25%

LLCI, lower limit of the 95% confidence interval; ULCI, upper limit of the 95% confidence interval. The proportion of the total effect (%) indicates the variance explained by the direct and indirect paths.

The results indicate that the sense of emptiness has a partial mediating role in the relationship between professional identity and learning burnout. Specifically, a lower level of professional identity is associated with higher levels of learning burnout through an increased sense of emptiness. The mediating effect accounts for 48.25% of the total effect, and the corresponding path coefficients are shown in Figure 2.

### 4.4 Testing the moderating effect of career calling

To examine the moderating role of career calling in the proposed model, the PROCESS macro (Model 14) was applied. The results indicated that career calling significantly moderated the relationship between emptiness and learning burnout (*β* = 0.0663, *p* = 0.014). Specifically, the strength of the positive association between emptiness and learning burnout varied across different levels of career calling: The stronger the level of career calling was, the stronger the positive predictive effect of emptiness on learning burnout (see Table 4).

**Table 4:** Moderation analysis of career calling on the relationship between feelings of emptiness and academic burnout.

Level of career calling	Indirect effect	Boot SE	Boot LLCI	Boot ULCI
Low	0.3511	0.0303	0.2915	0.4106
Medium	0.3916	0.0250	0.3425	0.4407
High	0.4322	0.0295	0.3742	0.4902

Boot LLCI, lower limit of the 95% bootstrap confidence interval; Boot ULCI, upper limit of the 95% bootstrap confidence interval.

However, the confidence interval for moderated mediation [w = −0.0462, 95% CI (−0.1106, 0.0033)] included zero, suggesting that although career calling intensified the direct effect of emptiness on learning burnout, it did not significantly alter the overall strength of the indirect pathway “professional identity → emptiness → learning burnout.” Furthermore, the direct effect of career calling on learning burnout was not significant (*β* = −0.0235, *p* = 0.4505), indicating that individuals with a greater sense of calling may experience greater psychological exhaustion when confronted with emptiness.

To provide a clearer interpretation of the moderating effect, participants were categorized into high and low career calling groups on the basis of one standard deviation above and below the mean of their career calling scores, respectively. A simple slope analysis was subsequently performed to further examine the predictive effect of emptiness on learning burnout at different levels of career calling.

**Figure 3:**
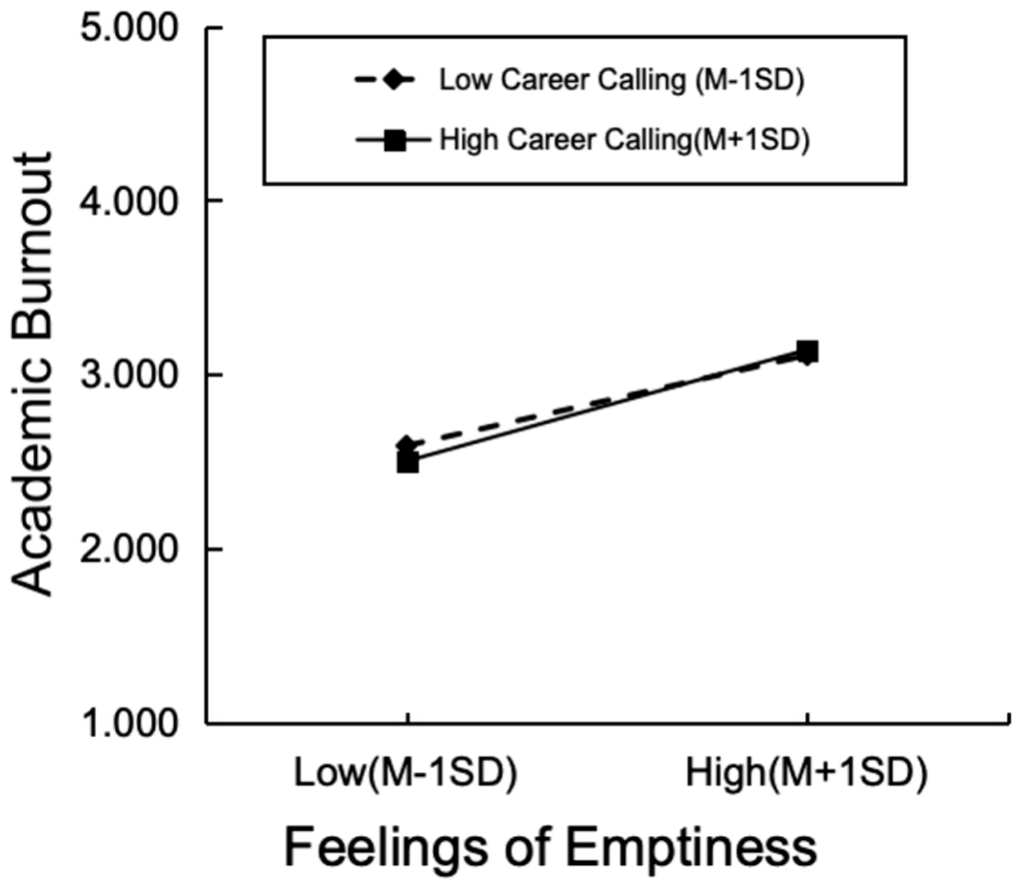
Conditional effects of feelings of emptiness on academic burnout at different levels of career calling.

The results revealed that when career calling was high, emptiness significantly and positively predicted learning burnout (*β* = 0.4322, *p* < 0.001). Similarly, when career calling was low, emptiness significantly and positively predicted learning burnout (*β* = 0.3511, *p* < 0.001). However, the magnitude of these effects differed, indicating that the interaction term between career calling and emptiness significantly predicted learning burnout (see Figure 3).

In summary, while career calling did not significantly alter the overall effect of professional identity on learning burnout, it did amplify the pathway between emptiness and learning burnout. For college students with a high level of career calling, experiences of emptiness may induce stronger psychological conflicts and consequently lead to greater learning burnout. These findings imply that for students with high levels of career calling, particular attention should be given to their feelings of emptiness to prevent intensified burnout resulting from the dissonance between strong career calling and a sense of inner emptiness.

### 4.5 Hypothesis testing

The path coefficients of the model were estimated using the PROCESS macro (see Figure 2). The results revealed that professional identity was a significant negative predictor of learning burnout (*b* = −0.5702, *p* < 0.001), thus supporting H1. Professional identity also had a significant negative effect on emptiness (*b* = −0.6971, *p* < 0.001), providing support for H2a. Emptiness, in turn, significantly and positively predicted learning burnout (*b* = 0.3946, *p* < 0.001), supporting H2b.

The direct effect of professional identity on learning burnout remained significant (*b* = −0.2951, *p* < 0.001), indicating that emptiness partially mediated this relationship. The bootstrap test further confirmed the significance of this mediating effect, with an indirect effect of −0.2751 and a 95% confidence interval of [−0.3225, –0.2279], which did not include zero, thus supporting H2c.

Moreover, the interaction term between emptiness and career calling had a significant moderating effect on learning burnout (*b* = 0.0663, *p* = 0.0140). However, the direction of moderation was contrary to the initial hypothesis. The results revealed that career calling amplified, rather than attenuated, the positive effect of emptiness on learning burnout. Specifically, a higher level of career calling was associated with a stronger positive relationship between emptiness and learning burnout. Therefore, H3 was not supported.

## 5 Discussion

This study was designed to explore the mechanism underlying the effect of professional identity on learning burnout among college students within a moderated mediation framework. Specifically, it examined the mediating role of a sense of emptiness and the moderating role of career calling. The findings contribute to a deeper understanding of the formation of learning burnout among college students and provide theoretical and practical implications for psychological support as well as learning interventions in higher education.

### 5.1 Discussion of the main variable results

First, professional identity significantly and negatively predicted learning burnout among college students. The significant negative correlation between professional identity and learning burnout (*r* = −0.609, *p* < 0.01), along with a path coefficient of −0.2951 (*p* < 0.001), indicates that students with higher levels of professional identity are less likely to experience learning burnout. According to self-determination theory (Ryan and Deci, 2000), individual behavior is driven by intrinsic motivation. When college students identify strongly with their chosen major, their intrinsic motivation to learn is activated, which promotes positive learning engagement and reduces burnout behaviors (Güngör and Sari, 2022). Furthermore, higher professional identity enhances students’ sense of self-efficacy, enabling them to experience a greater sense of control and achievement in their studies, further mitigating the risk of learning burnout (Zou et al., 2024).

Second, professional identity indirectly affects learning burnout by reducing the sense of emptiness. Mediation analysis revealed that emptiness partially mediated the relationship between professional identity and learning burnout [indirect effect = −0.2751, 95% CI (−0.3225, −0.2279)]. This finding indicates that higher professional identity is associated with lower levels of emptiness, which in turn reduces learning burnout. Students with a strong professional identity often possess clearer purpose and meaning in their academic pursuits, enabling them to experience a stronger sense of belonging and value in their studies (Yukhymenko-Lescroart, 2024), thereby feeling less empty. Conversely, students with low professional identity tend to lack interest in their academic field and have an unclear sense of direction, which makes them more susceptible to confusion, helplessness, and ultimately a stronger sense of emptiness (Hang et al., 2025; Wu et al., 2024).

According to Frankl’s (1984) logotherapy theory, individuals who fail to find meaning or purpose in life are prone to “existential emptiness.” To compensate, they may engage in maladaptive behaviors such as excessive sleeping, alcohol abuse, or internet addiction, which in turn exacerbate learning burnout. Therefore, along the causal chain “professional identity → sense of emptiness → learning burnout,” low professional identity can lead to goal loss and meaninglessness, trigger feelings of emptiness, and further increase burnout through maladaptive coping behaviors.

Third, career calling positively moderated the effect of emptiness on learning burnout. The interaction term between emptiness and career calling significantly predicted learning burnout (*b* = 0.0663, *p* = 0.0140), suggesting that career calling strengthened—rather than weakened—the positive association between emptiness and learning burnout. Contrary to the initial hypothesis, high levels of career calling did not buffer the negative impact of emptiness but rather amplified it. These findings can be interpreted through cognitive dissonance theory (Festinger, 1972). Individuals with high levels of career calling tend to hold lofty professional ideals and strong role expectations (Wrzesniewski et al., 1997). When they experience a sense of emptiness during their studies, the discrepancy between ideals and reality induces cognitive dissonance, leading to psychological discomfort. To reduce this discomfort, they may redirect their negative emotions toward the learning context, attributing dissatisfaction to their courses, teachers, or academic tasks. This process intensifies feelings of helplessness, detachment, and cynicism, ultimately exacerbating learning burnout. In other words, under conditions of emptiness, high levels of career calling amplify the conflict between ideals and reality, thereby increasing the risk of learning burnout.

### 5.2 Theoretical contributions

#### 5.2.1 Uncovering a new mechanism of sense of emptiness in the relationship between professional identity and learning burnout

Although prior research has shown that a sense of emptiness may influence learning burnout (Wang et al., 2025), no studies have systematically examined the specific mechanism through which emptiness affects the relationship between professional identity and learning burnout. Through empirical analyses, this study reveals that a sense of emptiness partially mediates this relationship. Specifically, professional identity reduces university students’ sense of emptiness, which in turn alleviates their learning burnout. This finding not only fills a gap in the literature but also deepens the understanding of the underlying pathways through which professional identity affects learning burnout. Introducing sense of emptiness as a mediating variable enables a more comprehensive explanation of how professional identity indirectly shapes learning behavior (i.e., learning burnout) by influencing students’ psychological states (i.e., emptiness), thereby offering new theoretical directions for future research.

#### 5.2.2 Clarifying the boundary conditions of career calling in the relationship between a sense of emptiness and learning burnout

Previous research has focused primarily on the facilitating role of career calling in promoting positive individual outcomes such as work engagement and career satisfaction (Shang et al., 2022; Wells et al., 2023) but has largely overlooked its potential moderating function in negative psychological and behavioral processes such as learning burnout. This study is the first to investigate the moderating role of career calling in the relationship between a sense of emptiness and learning burnout, revealing that career calling does not buffer the adverse effects of emptiness as expected; instead, it amplifies this effect. These unexpected findings challenge conventional assumptions and highlight the complex and context-dependent nature of career calling. This suggests that while the positive functions of career calling should be acknowledged, attention must also be given to its potential detrimental effects under certain psychological conditions—such as heightened feelings of emptiness. This insight provides important boundary conditions for future research on career calling.

### 5.3 Practical implications

The findings suggest that mitigating learning burnout among college students requires a multifaceted approach centered on enhancing professional identity, addressing feelings of emptiness, and appropriately guiding career calling.

#### 5.3.1 Establishing a systemic educational support mechanism centering on the development of professional identity

At the administrative level, institutions are encouraged to refine professional positioning, clarify training objectives, and strengthen the alignment between curricula and industry demands, thereby creating a “structured pathway for professional identity development.” Such efforts help students accurately understand the value of their discipline and the direction of their academic and career pursuits. At the instructional level, faculty are advised to adopt meaning-oriented pedagogical approaches—such as discipline-specific case analysis, situational practice, and authentic professional role experiences—to deepen students’ understanding of the meaning inherent in learning and enhance the satisfaction of their autonomy needs. Moreover, universities should offer professional identity workshops, career exploration courses, and faculty mentoring programs to facilitate value clarification and long-term career goal planning. These initiatives support students in constructing an integrated cognitive structure that links self-concept, academic major, and future profession, thereby reducing the risk of learning burnout.

#### 5.3.2 Integrating voidness identification and meaning enhancement into university psychological support systems

Universities should incorporate the identification of students’ sense of voidness and the enhancement of meaning in life as core components of their psychological support systems to address learning burnout at its source. Specifically, institutions may embed voidness-related indicators into routine psychological assessments and early warning systems to detect students who may be experiencing potential adjustment difficulties at an early stage. At the level of psychological services, universities are encouraged to implement structured meaning-oriented interventions—such as life education courses, goal-setting training, and meaning-making group programs—to help students reconstruct value frameworks, clarify developmental goals, and strengthen their sense of social connectedness. In addition, promoting student involvement in campus organizations, volunteer activities, and project-based learning can effectively increase relational support and a sense of contribution, thereby mitigating the detrimental impact of voidness on learning engagement. These efforts collectively foster a positive cycle that supports psychological fulfillment and academic adaptation.

#### 5.3.3 Guiding career calling in a scientifically informed manner to prevent mission overload-related psychological risks

Educators should recognize the “double-edged nature” of career calling and adopt theory-driven strategies to help students maintain a healthy balance between mission-driven motivation and psychological well-being. First, students with strong career calling should be supported in conducting expectation–reality calibrations. By providing updated industry information, career development courses, and mentoring support, universities can guide students in setting attainable professional goals and reduce the degree of cognitive dissonance caused by overly idealized expectations. Second, resilience-building and sustainable engagement principles should be embedded into talent development systems. Interventions such as frustration tolerance training, emotion-regulation counseling, and goal-decomposition strategies can strengthen students’ ability to manage internal resources and cope with setbacks effectively.

Finally, mental health practitioners should identify and intervene in latent risks such as “mission pressure” and “mission anxiety.” Through cognitive restructuring techniques and value-clarification counseling, practitioners can help students sustain psychological resilience and self-care while pursuing professional meaning. Such efforts ultimately reduce the likelihood of burnout and promote healthier, more sustainable career development trajectories.

## 6 Limitations and future directions

Despite its contributions, several limitations of this study should be acknowledged.

First, the adoption of a cross-sectional design precluded the examination of the dynamic evolution of professional identity and its long-term influence on learning burnout. Consequently, the causal relationships identified in this work should be interpreted with caution and further validated through longitudinal or experimental research.

Second, the relationship between professional identity and learning burnout may be influenced by other additional potential factors, including self-efficacy, social support, or learning motivation. Future research should employ longitudinal or experimental designs to explore diverse mediation and moderation mechanisms and to test the generalizability and robustness of the current findings.

Third, this study focused on Chinese university students, which provides valuable insights into learning-related behaviors within a specific cultural context. However, variations in cultural norms, educational systems, and economic development across countries and regions may shape students’ levels of professional identity and their experiences of learning burnout. Future research could incorporate contextual factors from diverse cultural, political, and economic settings and conduct cross-cultural comparative studies to enhance the generalizability and contextual robustness of the findings.

## 7 Conclusion

The impact of professional identity on learning burnout among university students was systematically examined in this study, and the mediating role of emptiness and the moderating role of career calling were further tested. The findings indicate that professional identity not only directly affects students’ levels of learning burnout but also indirectly affects burnout by influencing their sense of meaning and psychological fulfillment. Moreover, career calling—functioning as a key individual characteristic—amplifies the effect of emptiness on learning burnout. Together, these results offer empirical evidence and novel theoretical insights into the mechanisms underlying learning burnout and the pathways through which it may be alleviated.

First, the pivotal role of professional identity in mitigating learning burnout was confirmed in this study. Students with higher levels of professional identity are more likely to experience a sense of value and purpose in their academic pursuits, which in turn enhances their learning motivation and positive emotions, thereby significantly reducing burnout. These findings align with the core propositions of self-determination theory, which posits that intrinsic motivation and a sense of personal value function as essential resources for sustaining engagement and supporting psychological well-being.

Second, a sense of emptiness was identified as a key psychological mechanism linking professional identity and learning burnout. Students with lower levels of professional identity are more prone to experience a lack of meaning and psychological void, which can trigger negative emotions, compensatory behaviors, and academic disengagement, ultimately increasing the risk of learning burnout. The significant mediating effect of emptiness suggests that interventions targeting learning burnout should not only address students’ observable learning behaviors and emotional symptoms but also focus on fostering a sense of meaning and personal value.

Third, career calling significantly moderates the relationship between a sense of emptiness and learning burnout. Contrary to expectations, a high level of career calling did not buffer the negative effects of emptiness; rather, it amplified the impact of emptiness on learning burnout. These findings suggest that students with strong career calling are more likely to experience pronounced discrepancies between ideals and reality when confronted with a lack of meaning, leading to increased academic stress and psychological exhaustion. The results reveal the “double-edged sword” nature of career calling, expanding the existing research that has predominantly emphasized its unidirectional positive effects.

In summary, this study highlights the interactive mechanisms among professional identity, a sense of emptiness, and career calling in the development of learning burnout. The findings provide important implications for universities seeking to enhance students’ professional adaptation, optimize learning experiences, and deliver targeted psychological support. Future research could further explore differential mechanisms across students from diverse backgrounds and employ longitudinal data to examine the dynamic causal relationships among variables, thereby deepening the understanding of the psychological processes underlying learning burnout and improving the effectiveness of intervention strategies.

## Data Availability

The raw data supporting the conclusions of this article will be made available by the authors, without undue reservation.

## Footnotes

1
www.wjx.cn
